# Roles of nitric oxide in adaptive response induced in zebrafish embryos *in vivo* by microbeam protons

**DOI:** 10.1093/jrr/rrt161

**Published:** 2014-03

**Authors:** Viann Wing Yan Choi, Candy Yuen Ping Ng, Alisa Kobayashi, Teruaki Konishi, Masakazu Oikawa, Shuk Han Cheng, Peter Kwan Ngok YU

**Affiliations:** 1 Department of Physics and Materials Science, City University of Hong Kong, Hong Kong; 2 Department of Technical Support and Development, National Institute of Radiological Sciences, 4-9-1 Anagawa, Inage, Chiba 263-8555, Japan; 3 Department of Biology and Chemistry, City University of Hong Kong, Hong Kong; 4 State Key Laboratory in Marine Pollution, City University of Hong Kong, Hong Kong

**Keywords:** protons, adaptive response, nitric oxide, zebrafish embryos

## Abstract

Radioadaptive response (RAR) was successfully induced in dechorionated (5 h post-fertilization, hpf) embryos of the zebrafish, *Danio rerio*, by 3.4 MeV protons from the microbeam irradiation facility (Single-Particle Irradiation System to Cell, acronym as SPICE) [ 1] at the National Institute of Radiological Sciences (NIRS), against a challenging exposure of 2 Gy of X-ray irradiation at 10 hpf. The RAR induction was corroborated by reduced apoptotic signals at 25 hpf revealed through terminal dUTP transferase-mediated nick end-labeling assay. If *de novo* synthesis of factors was required for RAR induction, these should have already been synthesized at 5 h after the priming dose.

Application of a nitric oxide scavenger 2-(4-Carboxyphenyl)-4,4,5,5-tetramethylimidazoline-1-oxyl-3-oxide (cPTIO) to the medium at 0, 1, 2, 3 or 5 h after application of priming exposure significantly suppressed RAR. The suppression of RAR with the application of cPTIO to the medium at 5 h after the priming dose irradiation, where *de novo* synthesis of factors should have been completed, suggested that NO scavenging impaired the repair machineries in the bystander cells. The suppression of RAR with the application of cPTIO to the medium at earlier than 5 h after the priming dose irradiation could be explained by the scavenging of bystander NO signals in the medium and thus deterring the *de novo* synthesis of factors.
